# Insect circadian plasticity as a proposed target for the expression of parasite extended phenotypes

**DOI:** 10.1038/s44323-025-00046-0

**Published:** 2025-08-01

**Authors:** Joana Dopp, Charissa de Bekker

**Affiliations:** Microbiology, Department of Biology, Utrecht University, Utrecht, the Netherlands

**Keywords:** Circadian regulation, Diseases

## Abstract

Both parasite manipulation of host behavior and the roles of circadian clocks in infectious disease are not well understood. However, studies into parasite-manipulated insects suggest that host rhythms are altered at different levels of biological organization. Here, we discuss this hypothesis in the context of circadian plasticity. We argue that striking overlap between manipulation mechanisms and plastic functioning of the insect clock exists across independently evolved parasite-host systems. As such, investigating parasitic behavioral manipulation provides an opportunity to better understand circadian plasticity and how infection and clocks intersect across taxa.

## Introduction

Environmental factors including light, temperature, food availability, social dynamics and predation threat fluctuate across daily, monthly, seasonal and annual cycles. To anticipate these cycles, organisms throughout nature have evolved rhythmic physiology and behaviors tailored to their unique ecological niches and challenges^1,2^. This includes (endo)parasites: organisms that invade and take advantage of the resources of another organism - their host. Parasites need to coordinate their activities to external abiotic fluctuations and fluctuations within the host. For instance, daily rhythms in host immune responses have provided selection pressures for parasites to time host invasion and circumvent phases of high immunity, thereby increasing infection success^3^. Exploring the roles of biological clocks in parasite-host interactions and co-evolution could, therefore, contribute to our understanding of these ecologically ubiquitous interactions and improve treatment for clinically and agriculturally relevant infections.

**Fig. 1:**
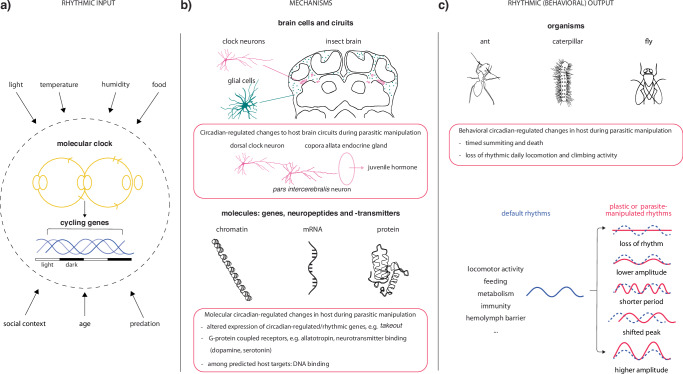
Host circadian plasticity as a target for parasitic manipulation of behavior. A visual summary of the hypothesis that parasites manipulate host behavior by targeting their circadian plasticity, explained at three levels of biological organization: molecular, cellular and behavioral phenotypes. **a** The molecular clock consists of a handful of interacting core circadian genes that create robust transcription-translation feedback loops (yellow). These interactions repeat in a ~24 h rhythm and entrain many downstream genes to cycle in their expression levels (blue). The dotted circle symbolizes the surrounding environment of an organism, which contains multiple factors that can act as predictable 24 h Zeitgebers and present disturbances that require some level of clock plasticity. **b** Cellular and molecular mechanisms of circadian plasticity. Red boxes include examples of parasitic manipulation on both levels. Cell bodies of clock neurons (magenta) and glial cells (teal) are illustrated in an ant brain slice (adapted from^90^). **c** Examples of rhythmic output that can be measured at the behavioral level in insects (e.g. ant, caterpillar and fly) and how these rhythms may change because of environmental factors or parasitic manipulation.

Organismic rhythms are driven by endogenous, robust molecular clocks. These clocks consist of negative transcription-translation feedback loops, that entrain to cycling environmental factors (i.e., Zeitgebers) and regulate oscillations in gene expression and protein production^4^ (Fig. 1). Since circadian clocks and their outputs exist to anticipate predictable 24 h rhythms, they should also be somewhat plastic to allow for responses to both gradual and acute changes in the environment (Fig. 1). This plasticity can be observed when matters such as food availability and social context are involved. For instance, mosquitoes, exhibit periodicity in their flight and blood-feeding activity^5^, which are modulated by temperature and the mosquito’s nutritional status^6–8^. Similarly, nocturnal mice gradually adjust their active phase when food is experimentally restricted to the daytime, being able to quickly reverse again when these restrictions are lifted^9,10^. Moreover, the honeybee *Apis mellifera*, uses social cues related to food availability to synchronize circadian foraging times among colony members^11^. Flexible rhythmicity is also observed in organisms as they age. When aging, adult humans typically transition to an earlier chronotype^12^ while worker ants and bees shift from seemingly aperiodic activity to 24 h rhythms to fulfil respective nurse and forager caste roles in the colony^13^. However, when sudden disruptions in colony organization or changes in social context occur, foragers can also quickly become nurses again, and vice versa^14^. Additionally, endoparasites, such as microfilaria and malaria parasites, show adaptive flexibility in their transmission timing to align to the daily activity peaks of their specific mosquito vectors^15–17^.

While circadian plasticity provides obvious fitness benefits, it could also keep the door open for unwanted modulation and corruption. Parasites cannot only adapt timed strategies to match host activity and circumvent heightened immune response, but in cases of prolonged co-evolution also manipulate and weaken this response^18^. Some parasites even enhance the intimate relationship with their host by adaptively manipulating their morphological or behavioral phenotypes to benefit parasite fitness. These extended behavioral phenotypes often involve abnormal daily timing, begging the question whether circadian clocks play a prominent role in parasite manipulation of host behavior. While the molecular mechanisms that underlie this phenomenon are currently not clear, hijacking an existing, somewhat flexible host system that modulates multiple behaviors provides a potential opportunity for enhancing parasite fitness. Here, we make a case for the host circadian system to be a putative, convergently evolved target for manipulating parasites, because a) it regulates many downstream behaviors and b) it is plastic. We do so by discussing the topics of circadian plasticity and parasitic behavioral manipulation at different levels of biological organization: 1) organismic, 2) cellular, and 3) molecular (Fig. 1). We hereby mostly focus on insects and their manipulating parasites since these interactions have currently been studied most extensively in the context of the circadian clock.

## Altered circadian timing: a convergently evolved parasite extended phenotype

To optimize their fitness, some parasites act through the host body to influence rhythmic activity patterns (Fig. 1). Prime examples involve fungi that invade and manipulate insect hosts, including the behavioral changes observed in ‘zombie’ ants infected by *Ophiocordyceps*. Infected ants lose their otherwise circadian foraging activity, become hyperactive and walk around seemingly directionless throughout the light/dark cycle. This is coupled with a loss of communication and collaboration with nest mates^19,20^. These behavioral changes are thought to promote fungal fitness by minimizing host interactions with nest mates that attack infected ants as a social immunity response threatening completion of the fungal life cycle^21^.

After two to three weeks of infection, the ant shifts from directionless arrhythmic hyperactivity to targeted, timed climbing of vegetation close to its end of life^20,22^, referred to as summiting. In nature, summiting is concluded around solar noon when the ant attaches itself at an elevated position by biting into the vegetation with its mandibles^23^. Such timed summiting is also observed across other fungus-infected insects, including flies infected by *Entomophthorales*^24^. These fungi reside in a different phylum from *Ophiocordyceps* and induce summit behaviors around dusk. Though summiting is synchronized to different times of day for *Ophiocordyceps* and *Entomopthorales* fungi, the adaptive function appears to be similar: placing the insect host under environmental conditions that favor spore production and eventually, transmission. Increased humidity from dusk till dawn promotes the quick sprouting of infectious *Entomophthora* conidia from fresh fly cadavers^25^. Instead, for *Ophiocordyceps* the development of spores and their dispersal can take up to several months^26^. To assure an optimal microclimate that will both preserve the cadaver during that time and promote eventual fruiting body (spore-bearing structure) formation, *Ophiocordyceps*-infected ants attach themselves at very specific incident light levels under the canopy^26^. When light conditions in the field were experimentally altered, the production of *Ophiocordyceps* fruiting bodies was reduced^27^ and moving the cadaver itself completely halted development^28^. Summiting and subsequent death of infected ants occurring when sun intensity peaks might, therefore, promote fitness by facilitating the seeking of ideal conditions for spore formation. Following fungal spore dispersal is also timed, seemingly to coincide with the host’s peak activity to maximize transmission opportunities. *Ophiocordyceps* releases spores around dusk to infect nocturnal forager ants while *Entomophthora muscae* targets diurnal flies at dawn^29,30^. Taken together, these manipulating fungal insect parasites appear to have evolved to both anticipate the daily activity rhythms of healthy hosts and induce altered rhythms upon infection. Being able to modulate and anticipate host timing are likely parasite-adaptive traits since failure to produce infective propagules or spreading them at the wrong time would jeopardize transmission success and parasite survival.

Affecting host timing appears to play an adaptive role in non-fungal parasites as well. Baculoviruses that infect caterpillars show parallels with fungal extended phenotypes as they also increase locomotion activity and induce climbing behaviors in moth species such as *Lymantria dispar*^31^. The climbing behavior of these moth larvae is restricted to the nighttime to facilitate feeding, while inactivity during the day promotes survival by avoiding predation, parasitoids and overheating^32,33^. Baculovirus infection disrupts this daily climbing rhythm - larvae remain in their elevated positions where they eventually die and liquify. This elevation promotes transmission of infectious particles either by them raining down across a larger area of vegetation, or through transportation by birds that can spot the infected caterpillars more easily^31,34–36^. While this might suggest that timed extended phenotypes only occur in strictly terrestrial parasite-host systems that involve summiting, the life cycle of horsehair worms also involves timed host manipulation. These parasites induce terrestrial hosts such as crickets^37^ and mantids^38^ to jump into bodies of water. During the nighttime, infected hosts become attracted to reflective light coming from water puddles^39^. By sensing horizontally polarized light to distinguish water depth, hairworm-infected mantids specifically steer towards deeper, perennial ponds to increase the parasite’s mating chances^40^.

Though convergence in behavioral manipulation phenotypes exists across natural parasite-host systems, variation in these systems constitutes a challenge in understanding behavioral manipulation at an organismal level. Additionally, observations are inherently biased by the human perspective. It is difficult to imagine what senses beyond our own play determining roles in other species. As such, we likely overlook many extended behaviors relevant for specific ecological parasite niches. The challenge, therefore, lies in taking the host perspective to understand how its parasite found ways to manipulate it to its advantage^41^. Moreover, descriptions of extended phenotypes across species evoke questions about their underlying molecular causal mechanisms and whether those have convergently evolved. To explore this, the following two sections will outline our current knowledge on how plastic and manipulated circadian rhythms are regulated in the brain on 1) a cellular and 2) a molecular level (Fig. 1).

## Heterogeneous cellular networks facilitate plasticity of the circadian clock and rhythmic behavior

The basis of (adaptable) behavioral circadian rhythms lies in diverse and interconnected brain cells that keep time by expression of the molecular clock, entrain downstream cells and ultimately regulate cyclic behavior. The most extensive research into clock cellular network organization and how it relates to circadian plasticity has been conducted in *Drosophila*. The fly clock cellular network consists of 120 cells per hemisphere (only around 0.1% of a fly brain)^42^, and is divided into 15 lateral and 105 dorsal clock neurons (LNs and DNs). The smaller population of LNs has been the focal point of insect circadian research in the past since these cells generate endogenous rhythmicity regardless of external cues^43^. Additionally, small ventral LNs (sLNv) are easier to study than DNs, because they are fewer in number, anatomically distinct and easily targeted with functional genetic tools^42,44^. Only recently the heterogeneity within DNs has been characterized in terms of connectivity, morphology and gene expression based on connectome^42,44^ and single-cell transcriptomic work^45,46^. These high-resolution datasets open new possibilities to explore the functional roles of diverse DNs in regulating downstream rhythmic behavior. As they depend on external Zeitgebers, DNs modulate, rather than generate behavioral rhythms according to those environmental cues (as reviewed in refs. ^47–49^). Therefore, DNs appear most relevant for dynamically regulating the adaptation of rhythmic behavior in natural settings (Fig. 1).

The clock cell network primarily relies on paracrine signaling via neuropeptides to communicate to downstream targets and regulate animal behavior. Neuropeptides act slower but last longer than synaptic neurotransmitters, enabling dynamic adaptations to environmental conditions^50^. Examples include pigment-dispersing factor (PDF) and allatostatin, which is known to inhibit secretion of juvenile hormone (JH), an important insect hormone implicated in development and behavior across insect species^51–53^, including ant behavior manipulation^20^ and behavioral caste division^54,55^. The composition of these neuropeptides differs significantly between cell subtypes, contributing to functional heterogeneity within the circadian system^42,56^. Furthermore, different neuropeptides appear to regulate circadian behavior in flies as compared to mammals and even other insect species. This suggests that the mode of signaling – paracrine – may be more critical than the specific molecule itself^57^. One class of DN downstream targets are the ~80 neurosecretory cells^42,56^, which regulate many plastic circadian behaviors such as feeding. One heterogeneous subset of these neurosecretory cells is located in the *pars intercerebralis* (PI) region - the fly functional homolog of the mammalian hypothalamus. The PI acts as a relay between circadian DNs and two endocrine glands located around the aorta and gut^56^. Importantly, the neuropeptidergic and hormonal signaling pathways within the circadian clock network appear to be key outputs to the endocrine system. This puts the clock in control of a wide array of behaviors and physiology^42,56^ and would make it an effective pathway for parasitic manipulators to hijack.

Besides dorsal clock neurons, non-neuronal, abundant and diverse glial cells may contribute to circadian plasticity (Fig. 1). Glia respond to metabolic and immune stressors such as temperature changes or infections in a circadian-dependent manner^58,59^. Unlike other non-clock neurons, they do express circadian clock genes^60^ and have the highest number of cycling genes compared to other cell types^61^, highlighting their important role in regulating circadian processes. In flies, the blood-brain barrier – more accurately a hemolymph barrier formed by glia - increases permeability at nighttime^62^. This circadian plasticity of the barrier allows for the timed flux of hormones, affecting the sleep:wake cycle by stimulating lipid metabolism in a specific glial subtype^63^. Furthermore, plastic remodeling of circadian neurons is enabled by sphingolipid metabolism in glia^64^. These studies indicate that glial cells, like DN clock neurons, may sense environmental changes and flexibly shape circadian-driven output via hormonal and paracrine signaling. This makes the clock system plastic, but also vulnerable to parasitic manipulation.

## Parasite strategies to manipulate host circadian cellular networks

While much remains unknown about the various cells within the clock network and how they orchestrate downstream circadian behaviors, even less is understood about interactions between the circadian clock networks of two different organisms. This axis of integration likely occurs to some extent in every endoparasitic interaction in which parasite and host have their own molecular clock. However, it seems most pronounced in interactions where host circadian outputs are measurably altered (e.g., malaria^65^ and sleeping sickness^66^) or even manipulated. Such rhythmic disease phenotypes provide an opportunity to study how clock networks intersect with infection and give rise to altered (behavioral) outputs.

To achieve precise behavioral manipulation, parasites must employ strategies to infiltrate and affect brain cells while maintaining their functionality. This may occur either by physical invasion of brain tissue or by altering brain network activity through effector release. Evidence for the former has been described in *E. muscae*-infected *Drosophila*. *Entomophthora* was shown to occupy space around the axons of posterior dorsal clock neuron type 1 (DN1p) and the dendrites of neurosecretory PI neurons^67^. A subset of these dendrites connects to an endocrine gland, the corpora allata (CA), which synthesizes and releases JH. Given the known roles of these players, it was hypothesized that *Entomophthora* hijacks the function of DN1p clock neurons and downstream PI-CA connectivity. Indeed, silencing or activating subsets of DN1p or PI neurons in *Drosophila* affected summiting^67^. Knocking down diuretic hormone 31(DH31) and CNMamide (CNMa) in the same DN1p subsets also reduced summiting, indicating that DN1p uses these peptides for signaling. However, summiting was not significantly impaired in fly mutants for DH31 and CNMa receptors. While this raises questions about the signaling mechanism, this discrepancy may be due to the unspecific fly-wide receptor disruption employed in these studies. Notably, disrupting sLNv neurons did not elicit the summiting phenotype, suggesting that the endogenous clock is not targeted by the fungus^67^. These findings are consistent with the notion that DNs, and not sLNv cells, sense the environment and modulate downstream circadian processes. Moreover, this study found that the fly glial hemolymph barrier was more permeabilized in *Entomophthora-*infected flies, possibly to enable easy passage of fungal behavior-manipulating effector proteins. While the increased permeability could be due to general barrier destruction, it may also be explained by the parasite exploiting the circadian-dependent flexibility provided by clock neurons and glia. These hypotheses could be tested by increasing the temporal resolution in which infected flies are studied to characterize the timeline of circadian system disruption.

Contrary to *Entomophthora* invasion into infected fly brains, both light and electron microscopy of *Ophiocordyceps*-infected ants indicate that these fungi do not penetrate brain tissue but invade muscle instead^23,68,69^. Perhaps other cells, that regulate circadian rhythms more generally, are targeted to disrupt and change circadian processes. Alternatively, *Ophiocordyceps* could be secreting effector peptides, proteins and metabolites to interact with host clock or clock-controlled proteins. Recent multi-omics work on *Ophiocordyceps*-infected ants suggests that this might be the case. However, this exploratory work was done on whole ant heads without taking brain heterogeneity into account. More detailed cellular level studies to investigate the brains of parasite-manipulated insects that exhibit circadian phenotypes and in which the parasite does not actively invade the brain tissue, would fill this knowledge gap. Such work would not only connect organismal level extended phenotypes with molecular underpinnings but also shine a light on the regulation of circadian plasticity and how it gives rise to rhythmic behaviors in general.

## Molecular mechanisms of circadian clock plasticity

To better understand the molecular mechanisms underlying circadian plasticity and behavior manipulation, one can interrogate the genetic basis of organisms evolving and exploiting circadian plasticity to either adapt to changing environments or manipulate behavior of another organism. While canonical clock genes oscillate in synchrony between clock cell types, calcium activity peaks differently across those groups, which is facilitated by neuropeptide modulation. This asynchrony was hypothesized to underlie the generation of diverse downstream behavioral rhythmic outputs^70^. Furthermore, the phases and regulatory program of circadian genes vary between clock neurons and glia^61,71,72^ (Fig. 1). For example, the accessibility of *timeless* (*tim*) cis-regulatory regions differs between clock neurons and glial cells in *Drosophila*^72^. External cues also modulate the molecular clock. To adapt to extreme light conditions in high-latitude environments, *Drosophila* flies evolved a novel *tim* allele, *ls-tim* encoding both a longer and shorter TIM isoform (L-TIM and S-TIM)^73^. LS-TIM is less sensitive to light due to a weaker interaction with the circadian photoreceptor CRYPTOCHROME^74^. This reduced light sensitivity allows temperature cues to dominate circadian entrainment over light synchronization, thereby increasing fitness in environments with long photoperiods in the summer^75^. Furthermore, the cis-regulatory regions of neuropeptide-encoding gene *Pdf*, which regulates circadian locomotor rhythms, are different in globalist *Drosophila melanogaster* compared to *Drosophila sechellia*^76^. The latter species is endemic to the equatorial Seychelles Islands characterized by ~12:12 light:dark conditions throughout the year. Specifically, in *D. melanogaster pdf* RNA abundance dynamically adapts to different Light-Dark (LD) regimes and is overall higher compared to *D. sechellia*^76^. These RNA levels correlate with a green fluorescent reporter expression that was lower and more constant when driven by the *pdf* regulatory region of *D. sechellia* compared to the one of *D. melanogaster*. Moreover, reproductive success was reduced in *D. sechellia*, but not *D. melanogaster*, when exposed to an LD cycle with a long photoperiod. This further highlights the evolutionary pressure that likely molded differential behavioral plasticity between these species and suggests that affecting the interaction capacity of gene regulatory regions is a putative mechanism through which behavior could be manipulated.

Examples of natural timing variation, regulated by the molecular circadian clock, have also been described beyond flies. The behavior of the marine midge *Clunio marinus*, which is under circadian and circalunar control, shows different adaptations across strains. This observed variation was attributed to alternative splicing of CAMKII.1, a protein kinase that affects transcriptional activity of core clock genes *Clock (Clk)* and *Cycle (Cyc)*^77^. The ecologically relevant role of flexible circadian-controlled gene expression has also been measured in carpenter ants. Here, differences in oscillating gene expression have been implied to play a role in behavioral caste switching. Whole brains of *Camponotus floridanus* forager ants expressed three times as many genes with a circadian rhythm compared to nurse ant brains. However, many of these 24 h cycling genes in foragers do cycle at the third harmonic (i.e., every 8 h) in nurse brains, and do so in the same phase as the core clock gene *period* (*Per*)^78^. This plasticity in cycling gene expression correlates with, and likely regulates, the difference in circadian locomotor activity between nurse and forager ants. Furthermore, co-expressing genes that were differentially expressed between these behavioral castes, were connected to rhythmic modules in a gene network analysis^30^, which further strengthens the connection between flexibility in clock-regulation and behavioral plasticity (Fig. 1).

## Molecular mechanisms of parasites to manipulate host circadian rhythms

Parasites likely interfere with multiple gene networks as they manipulate a broad set of host behaviors into a stereotyped sequence of events. Computational protein-protein interaction predictions that tested *Ophiocordyceps* putative effector protein sequences against the carpenter ant host proteome corroborate this^79^. Overrepresentation analysis of putative binding partners of fungal effectors predicted that ant proteins involved in transcription and DNA binding could be significantly targeted by the fungus. This supports the hypothesis that host behavioral plasticity could indeed be modulated by parasites at the gene regulatory level. However, for this to be plausibly achieved through effector binding, evidence for effector entrance into the host cell and even nucleus would be necessary. This machine learning approach also predicted 33 ant G-protein coupled receptors (GPCRs) as targeted by 16 *Ophiocordyceps* proteins and peptides in a total of 71 interactions^79^. Of these receptors, the majority were functionally annotated to bind different neuropeptides and biogenic monoamines such as dopamine and serotonin. Metabolomics data further supports involvement of monoamine neurotransmitters, as these were dysregulated during *Ophiocordyceps* manipulation^80^. Another two GPCRs were annotated as ultraviolet and opsin blue light sensitive. Multiple of these GPCRs were also found to be expressed in a circadian manner in carpenter ant brains^78^. Furthermore, receptors of allatotropin, which is associated with circadian and JH regulation, were predicted to be targeted by manipulation. As such, *Ophiocordyceps* could manipulate timed behavior by affecting light and ligand sensitivity of these receptors through binding of effectors that either block or activate G-protein pathways. However, how the processes downstream of these GPCRs could give rise to changes in host (timed) behaviors is currently unclear.

Current molecular evidence for parasitic manipulation of host circadian clocks mostly comes from transcriptomics studies. Most rhythmically expressed genes in the whole heads of healthy carpenter ants lost their daily expression pattern about halfway during *Ophiocordyceps* infection^81^. Such vast genetic rhythmicity loss could underlie the arrhythmic behavior observed in infected ants at this infection time point^19^. The rhythmic expression of core clock genes *Clk* and *Per* were not among the genes affected. These clock genes were found to co-express in one of three ‘24 h rhythm’ modules of a genome-wide ant gene co-expression network analysis. While this module stayed intact during fungal infection, the other two rhythm modules identified in this network, that contained circadian-regulated genes, significantly lost connectivity^81^. Therefore, while core clock function in the host ant remains, perhaps to facilitate timed summiting behavior at the end of the infection cycle and/or internal synchrony, the parasite seemingly affects the expression of downstream otherwise rhythmic genes. In turn, this induces the parasite-adaptive arrhythmic phenotypes that help to circumvent social immune responses of the colony. This hypothesis is supported by the finding that many of the same genes that are differentially expressed during fungal manipulation of ant behavior are also differentially expressed between nurse and forager ants^30^. Moreover, these overlapping behavioral plasticity and manipulated ant genes resided in a network module that was found to be significantly correlated to one of the rhythm modules. This further indicates a parasitic strategy to adaptively manipulate host behavior by hijacking the existing circadian plasticity that facilitates ant caste division.

Nevertheless, it must be noted that circadian rhythms are affected by infections and immune system activation more generally^82^. Therefore, teasing apart circadian effects specific to host behavior manipulation and those generally induced by infection is necessary. For this purpose, the generalist fungal pathogen *Beauveria*, which does not adaptively alter its host’s behavior, has proven to be a useful control. Upon *Beauveria* infection, far less ant host genes lost their rhythmic expression compared to *Ophiocordyceps* infections. Connectivity in rhythm network modules was also preserved^81^, and *Beauveria-*infected ants still showed significant daily activity rhythms^19^. However, both rhythmic ant host genes and behavioral rhythms demonstrated a phase shift in *Beauveria-*infected ants compared to healthy controls^19,81^. This suggests that certain circadian changes in gene expression and behavior are perhaps general hallmarks of disease, while others are infection or manipulation-specific.

Though most specifically studied in *Ophiocordyceps*-ant interactions so far, examples of molecular clock involvement in host behavioral manipulation have also been reported from other parasite-host interactions. In baculovirus-infected lepidopteran larvae the expression levels of circadian-regulated and core clock genes, including *Per* and *tim*, were altered across several ZT time points^83^. Such differential expression of *tim* has also been observed in transcriptomics data from *Entomophthora*-infected flies^84^. Moreover, knock-out studies demonstrated that viral protein tyrosine phosphatase (PTP) plays a demonstrable role in the induction of hyperactive host behavior by some baculovirus strains^85,86^. These phosphatases are hypothesized to interact with insect host targets such as *foraging*, which is linked to light perception and the circadian clock. Additionally, JH levels and *takeout*, both implicated in circadian regulated (feeding) behavior across insect species^87,88^, could be targeted by baculovirus PTP^85^. These PTP targets may in turn regulate neurotransmitter, -peptide and hormone signaling, ultimately resulting in abnormal caterpillar behavior.

Transcriptomics data from *Ophiocordyceps-*infected ants also suggest a role for parasite PTPs, indicating the potential for convergently evolved molecular mechanisms underlying manipulation phenotypes. Several secreted fungal *ptp’*s were significantly higher expressed during manipulated summiting behavior^20,22^. In addition, the expression of one of these fungal *ptp’*s, as well as other putative behavior-manipulating fungal genes, oscillate with a 24 h rhythm^89^. This suggests that *Ophiocordyceps* regulates the production of at least some of its manipulation molecules in a circadian manner. The PTP-target *takeout* was also downregulated during behavior manipulation in *C. floridanus*. Similarly, genes involved in JH production and degradation were dysregulated in *Ophiocordyceps*-infected ants^20^ and *E. muscae-infected* flies^84^, which in *E. muscae* infections has been linked to the circadian system as discussed above. It is yet to be seen if something similar is happening in fungus-infected ants and baculovirus-infected caterpillars as well. However, current hypotheses allude to this, which suggests that molecular clock processes that underlie behavioral plasticity and manipulation are conserved across insect hosts.

## Conclusion

Insect circadian plasticity is manifested across levels of biological organization – from behavioral phenotypes, down to cell circuits and gene expression. Studies investigating the widespread phenomenon of parasitic behavioral manipulation across these levels suggest that this innate circadian plasticity could be exploited to modulate host physiology and behavior in ways that benefit parasite transmission. Timing-related extended phenotypes have been described across independently evolved manipulation systems, suggesting that similar circadian-related mechanisms gave rise to them. For example, both in *Ophiocordyceps*-infected ants and *E. muscae*-infected flies, neuropeptidergic and hormonal signals seemingly play key roles in behavior manipulation, while also being the main signaling mode of the circadian clock in general. Interestingly, these parallel findings emerged from analyzing different organizational levels and applying distinct methods. In flies, studies so far have focused on dissecting how different cell populations may communicate to produce the summiting phenotype. In contrast, in ants, different omics technologies were used to identify candidate molecules involved in summiting.

However, it comes to no surprise that results across independent experiments, labs and manipulation systems show some level of congruence. Comparative studies of the molecular clock in general have found similar clock genes and feedback loop principles conserved across the animal kingdom. Following the pioneering study in flies that identified the first circadian gene *Per*, immunohistochemical studies have identified *Per*+ central pacemakers in other insects, including cockroaches, crickets, beetles, moths and eusocial insects like honeybees and ants^43,57,90,91^. Overall cellular organizations and circadian oscillations of clock gene expression and protein level are similar. Though species-specific differences that may have evolved to meet differences in ecological needs, also exist^57^. As such, parasite taxa that are on independent evolutionary trajectories towards host behavioral manipulation as a strategy to increase fitness are likely presented with similar selection pressures and opportunities to overcome them. Comparable host circadian rhythms would present similar selection pressures to which parasites must adapt, and circadian plasticity would provide a similar backdoor into hijacking them, leading to convergent parasite mechanisms. Such taxa-wide convergence in infection mechanisms is also seen in other aspects of parasite-host interactions besides manipulation^92^. Therefore, unraveling what role circadian clock plasticity plays in one manipulative parasite-host system might provide testable hypotheses for others and push the entire research field forward. Moreover, these manipulation systems with clear rhythmic disease phenotypes provide an opportunity to better understand the intersection between biological clocks and infectious diseases.

Taken together, we advocate for more comparative studies across manipulation systems and integration of the largely separated fields of chrono-/neurobiology, infection biology and behavioral ecology, to achieve a comprehensive understanding of behavior-manipulation mechanisms. Ecological field studies provide the opportunity to observe, record, and place (altered) rhythmic phenotypes into evolutionary context. However, such studies are prone to environmental variability that possibly masks biological effects and complicates addressing mechanistic questions. In contrast, molecular work explores biological questions by targeting detailed cellular and molecular processes in controlled conditions but typically lacks a discussion on the functional ecological relevance of these mechanisms. The trade-off between ecological relevance and mechanistic understanding may be overcome by applying various –omics technologies and functional studies under laboratory settings that carefully mimic natural conditions informed by ecological surveys. Many molecular techniques can be used with high cellular resolution, e.g. sequencing across single cells in a tissue. Such resolution may prove useful in future studies of neural mechanisms of behavior-manipulation, considering that only specific clock sub-populations appear relevant in manipulated flies^67^ and that the clock cellular network is highly heterogenous in general. Analyzing the cellular diversity of clock neurons will be important in the future for uncovering its functional relevance to generate circadian plasticity across different ecological contexts. The natural experiment that behavior-manipulating parasites provide might prove useful in this endeavor while unraveling the cells and genes involved in parasite-induced changes in host rhythmicity will in turn reveal their functions under normal circadian conditions.

## Data Availability

No datasets were generated or analyzed during the current study.
